# A Prospective Comparative Study on Cardiac Alterations After Surgery and Drug Treatment of Primary Aldosteronism

**DOI:** 10.3389/fendo.2021.770711

**Published:** 2021-11-11

**Authors:** Yi-Lin Chen, Ting-Yan Xu, Jian-Zhong Xu, Li-Min Zhu, Yan Li, Ji-Guang Wang

**Affiliations:** Department of Cardiovascular Medicine, National Research Center for Translational Medicine, Centre for Cardiovascular Evaluations, Shanghai Key Laboratory of Hypertension, The Shanghai Institute of Hypertension, Department of Hypertension, Ruijin Hospital, Shanghai Jiaotong University School of Medicine, Shanghai, China

**Keywords:** primary aldosteronism, adrenalectomy, mineralocorticoid receptor antagonist, left ventricular pressure-strain loop, left ventricular mass index, left atrial volume index

## Abstract

**Background:**

Current guideline recommends both surgery and drug treatment for primary aldosteronism. Treatment effects on the cardiac structure and function remain under investigation.

**Objective:**

We performed a prospective study in patients with primary aldosteronism to compare effects of surgery and drug treatment on the cardiac structure and function as assessed by the left ventricular (LV) pressure-strain loop, a novel echocardiographic technique that incorporates myocardial deformation and LV pressure.

**Methods:**

Our study included 39 and 28 patients treated with surgery and a mineralocorticoid antagonist, respectively. We performed conventional and speckle tracking echocardiography at baseline and 3 and 6 months of follow-up.

**Results:**

During follow-up, both surgery and drug treatment normalized serum potassium concentration and significantly reduced blood pressure. Both treatments significantly and similarly decreased LV mass index and left atrial volume index. However, only in the surgery group did global wasted work significantly decrease (200.8 ± 86.7 at baseline vs. 142.1 ± 58.1 mmHg% at 6 months) and global work efficiency (91.5 ± 3.1 *vs*. 93.6 ± 2.3%) and global longitudinal strain (−18.3 ± 2.7 *vs*. −19.2 ± 1.9%) significantly (*p* < 0.01) increase at 6 months of follow-up. The corresponding differences from the changes in the drug treatment group were 39.5 mmHg% (95% CI, 17.1, 62.0 mmHg%), −1.64% (95% CI, −2.56, −0.71%), and −0.85% (95% CI, −1.51, −0.20%), respectively. In addition, the changes in global wasted work at 6 months of follow-up was significantly correlated with that in 24-h urinary aldosterone excretion in the drug treatment group (*r* = 0.54) and two groups combined (*r* = 0.55), but not the surgery group.

**Conclusion:**

In spite of similar serum potassium normalization and blood pressure control, surgical removal of an adrenal gland, but not mineralocorticoid receptor antagonism, showed early improvement in cardiac function.

## Introduction

Primary aldosteronism is a common cause of secondary hypertension, with a prevalence ranging from 5% to 15% in hypertensive patients seen in various clinical settings (1, 2). Previous studies have shown that aldosterone hypersecretion in the adrenal glands may induce alterations in cardiac structure and function and increase cardiovascular morbidity and mortality (3) *via* increasing myocardial fibrosis independent of blood pressure (4). Current clinical guidelines recommend adrenalectomy for unilateral primary aldosteronism and medical treatment for bilateral lesions or those who are unwilling to undergo surgery (5). Several previous studies have demonstrated that these treatments, especially adrenalectomy, may reverse the cardiac structural alterations such as left ventricular hypertrophy (6, 7). However, there are very limited published data on the changes in cardiac function after surgery and drug treatment, especially with regard to the comparison between the two therapeutic modalities. A possible reason is probably the difficulty in the detection of early cardiac alterations with the conventional standard echocardiography.

Global longitudinal strain based on speckle tracking echocardiography is now recommended to evaluate early cardiac functional performance in addition to the traditional left ventricular ejection fraction in clinical practice according to current guidelines (8). However, the results of recent studies, on the other hand, suggested that this strain parameter was still load-dependent (9). Non-invasive left ventricular pressure-strain loop provides a novel technique to quantify myocardial function by incorporating measurements of myocardial deformation and left ventricular pressure (10, 11). This technique has been recently applied in clinical research and showed potential advantages over conventional global longitudinal strain in cardiac evaluations, as it allows a better understanding of the relationship between left ventricular remodeling and increased wall stress under various loading conditions (12, 13). Indeed, a recent study showed that myocardial work index was significantly increased in stage 2 and 3 hypertension compared with healthy participants while global longitudinal strain was similar in the two groups (12). This new technique provides new insights into the evaluation of cardiac function. In the present prospective observational study, we investigated the changes in cardiac structure and function after surgery and drug treatment with this novel method in patients with primary aldosteronism.

## Methods

### Study Population

We enrolled 67 consecutive patients with hypertension and primary aldosteronism, who had been either surgically or medically treated in our inpatient ward from November 2018 to July 2020. All patients underwent a standardized diagnostic procedure. After mineralocorticoid receptor antagonists, angiotensin-converting enzyme inhibitors, angiotensin receptor blockers, β-blockers, and diuretics were withdrawn for at least 4 weeks, and plasma aldosterone concentration (PAC, in pg/ml) and plasma renin activity (PRA, in ng/ml/h) were measured after overnight fast in the supine and standing positions. If a ratio of PAC to PRA (Aldosterone-to-Renin Ratio, ARR) was higher than 240 pg/ml per ng/ml/h, a physiological saline infusion test was performed to confirm the diagnosis of primary aldosteronism. The cutoff value for the post-saline PAC was ≥100 pg/ml. An adrenal venous sampling procedure and an adrenal computed tomography imaging were performed for the subtyping of primary aldosteronism and lateralization in whom adrenalectomy was considered. All patients with aldosterone-producing adenoma underwent unilateral adrenalectomy after lateralization and had their diagnosis confirmed histopathologically (5, 14, 15). Patients were excluded if they had other forms of secondary hypertension, ischemic heart disease, valvular heart disease, cardiomyopathy, pacemaker implantation, atrial fibrillation, or suboptimal echocardiographic windows. The study protocol was approved by the Ethics Committee of Ruijin Hospital, Shanghai Jiao Tong University School of Medicine, Shanghai, China. All study participants provided informed written consent.

### Blood Pressure and Laboratory Measurements

Blood pressure was measured twice consecutively with a 30- to 60-s interval after at least 5 min rest in the sitting position using a validated automated blood pressure monitor (HEM-9200T or HEM-7051, Omron Healthcare, Kyoto, Japan). These two blood pressure readings were averaged for analysis. In the absence of an inter-arm blood pressure difference of 10 mmHg or more, all blood pressure measurements were performed on the left arm during the whole study.

PAC and PRA were measured by using radioimmunoassay according to the manufacturer’s instructions (Beckman Coulter Corp., Brea, CA, USA). The intra- and inter-assay coefficients of variation were 9.3% and 9.5%, respectively, for PAC and 10.1% and 10.2%, respectively, for PRA.

### Conventional Transthoracic Echocardiography

We performed 2D, M-mode, continuous-wave, pulsed-wave, and pulsed tissue Doppler measurements in the sinus rhythm using the Vivid E9 and E95 Echo-system (General Electric Medical Health, Milwaukee, WI, USA) equipped with an M5S transducer. The recording of echocardiography from baseline to follow-up was performed by the same experienced ultrasonographer who was blinded to the treatment to avoid any impact on measurements. Images of parasternal long-axis view were recorded to measure left ventricular dimensions and interventricular septal (IVS) and left ventricular posterior wall thickness (LVPW). Relative wall thickness (RWT) was calculated by the formula: (2 × LVPW)/left ventricular end-diastole diameter (LVEDD). Left ventricular mass was calculated using the formula: 0.8 × {1.04 × [(LVEDD + IVS + LVPW)^3^ − LVEDD^3^]} + 0.6, and indexed by body surface area (BSA) as left ventricular mass index (LVMI). Left ventricular ejection fraction (LVEF) was calculated by left ventricular end-diastole volume (LVEDV) and end-systole volume using the modified Biplane Simpson method. The left atrial size was represented by left atrial maximal volume measured at end-systole from four- and two-chamber views, and indexed by BSA as left atrial volume index (LAVI). Pulsed-wave Doppler of mitral valve inflow in the four-chamber view was recorded to measure the peak early (E) and atrial filling (A) velocity, and the ratio of E/A. E’ was measured as the average of the peak early diastolic velocity at the septal and lateral mitral annulus by pulsed tissue Doppler. Tei index was defined as the sum of isovolemic contraction time (IVCT) and relaxation time (IVRT) divided by ejection time (ET) *via* pulsed-wave Doppler (8).

### 2-Dimensional Speckle Tracking Echocardiography Analysis

Images from the apical four-, two-, and three-chamber views were recorded with a frame rate over 45 fps. Global longitudinal strain was quantified using a semi-automated function imaging (EchoPac, Version 203; General Electric Medical Health, Milwaukee, WI, USA). Automated tracking of myocardial motion was performed with the region of interest adjusted by correcting the endocardial border or width. Global longitudinal strain was calculated from the average of the peak systolic longitudinal strain of all 17 segments.

### Left Ventricular Pressure-Strain Loop Analysis

Myocardial work indices based on left ventricular pressure-strain loop were calculated through a combination of longitudinal strain and a non-invasively estimated left ventricular pressure curve. Peak systolic left ventricular pressure is assumed to be equal to the peak arterial pressure, which was the brachial cuff systolic pressure measured immediately prior to the echocardiography study. The software then constructed the left ventricular pressure-strain loop adjusted according to the duration of isovolemic and ejection phases defined by the valvular timing events. The following parameters were calculated: global myocardial work index (GWI) defined as the total work within the area of the left ventricular pressure-strain loop, global constructive work (GCW) defined as the work during segmental shortening in systole adding negative work during lengthening in isovolemic relaxation, global wasted work (GWW) defined as the work during lengthening in systole and shortening in isovolemic relaxation, and global work efficiency (GWE) defined as GCW divided by the sum of GCW and GWW (10, 16).

### Follow-Up

**Figure 1:**
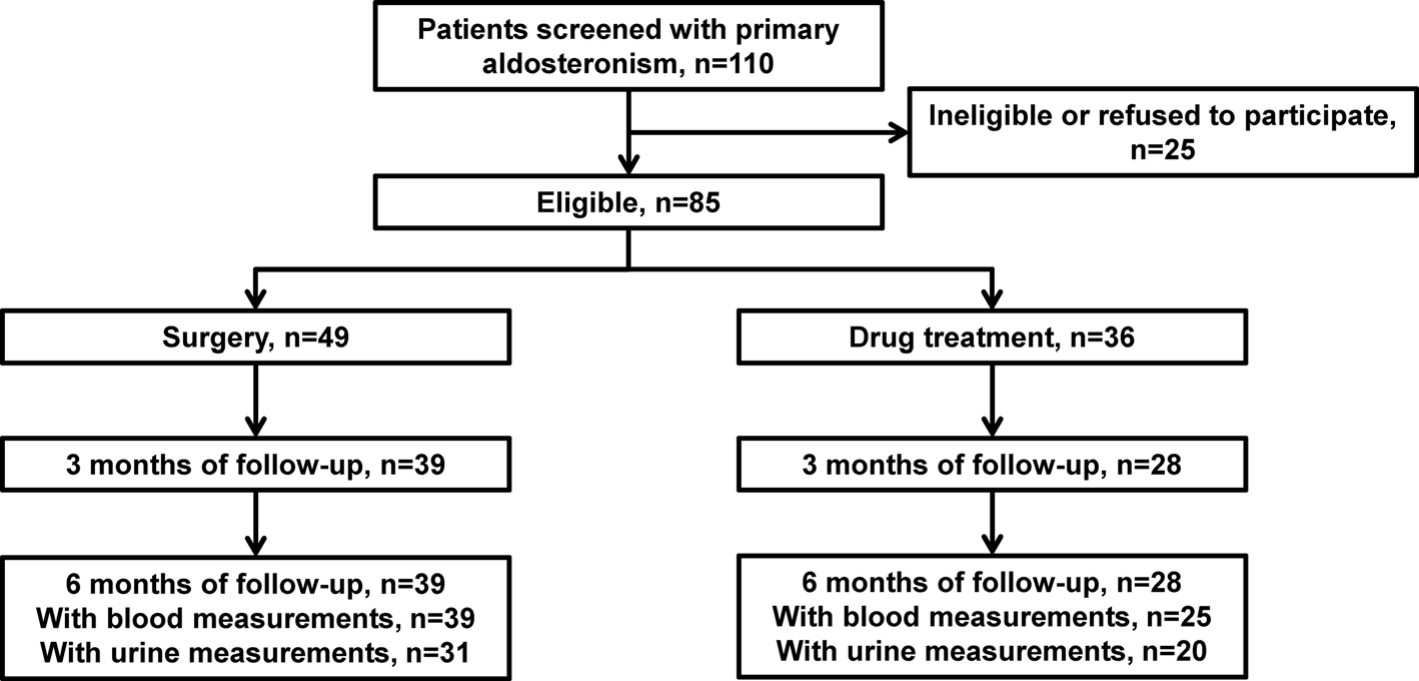
Flow of patients.

Thirty-nine patients underwent unilateral adrenalectomy, including 33 with aldosterone-producing adenoma and 6 with idiopathic hyperaldosteronism. Twenty-eight patients were treated with a mineralocorticoid receptor antagonist, including 13 patients with bilateral primary aldosteronism and 15 with the clinical requirement, but without intention of surgery. Standard transthoracic echocardiography was repeated at 3 and 6 months after discharge from the hospital. Biochemical measurements including PAC, PRA, serum potassium concentration, 24-h urinary aldosterone excretion, and 24-h urinary potassium concentration were performed between 3 and 6 months of follow-up (**Figure 1**).

### Intra- and Inter-Observer Variability

To evaluate intra- and inter-observer variability, 10 patients were randomly selected. The intra-observer variability was evaluated by analyzing the echocardiographic images at a different time by the same observer blinded to previous observations. The inter-observer variability was evaluated by analyzing the echocardiographic images of the same patients by a second observer blinded to the observations obtained by the first observer.

### Statistical Analysis

Statistical analyses were performed using the SPSS software version 22.0 (IBM Corp, Armonk, NY, USA). Continuous variables were expressed as mean ± SD or median (interquartile range) and analyzed using the Student’s *t*-test, paired sample *t*-test, Mann–Whitney *U* test, or Wilcoxon rank sum test, respectively. The Kolmogorov–Smirnov test was used to assess the distribution of continuous variables. Categorical variables were expressed as percentage and analyzed using the Chi-square test. Analysis of covariance was performed to calculate the least square mean changes and standard error, with baseline values as covariate and treatment group as a factor. One-way ANOVA with repeated measures followed by LSD test was performed for the between-group comparisons among measurements at baseline and 3 and 6 months of follow-up. Pearson correlation analysis was performed to study the relationship between the changes in myocardial work parameters and aldosterone levels. The intra- and inter-observer variability was evaluated by the use of the intra-class correlation coefficient (ICC). A *p*-value of less than 0.05 was considered statistically significant.

## Results

### Characteristics of Patients and Echocardiographic Measurements at Baseline

**Table 1** shows the demographic, clinical characteristics, and echocardiographic measurements at baseline in patients treated with surgery and antihypertensive drugs. The two groups of patients were comparable in age (49.1 ± 10.7 years, *p* = 0.830) and sex distributions (70.5% of men, *p* = 0.286), and in most of the clinical characteristics, such as body mass index (26.2 ± 3.0 kg/m^2^, *p* = 0.175), systolic and diastolic blood pressure (146.1 ± 13.6/85.2 ± 8.9 mmHg, *p* = 0.306 and 0.576), heart rate (67.5 ± 9.3 beats/min, *p* = 0.597), and the number of antihypertensive medications [2.0 (2.0, 3.0), *p* = 0.410]. They also had similar plasma aldosterone level (*p* = 0.469), urinary potassium level (*p* = 0.178), and renal function (*p* = 0.077). However, patients in the surgery group had a significantly lower PRA (*p* < 0.001) and serum potassium concentration (*p* = 0.036) and higher urinary aldosterone level (*p* = 0.021). During hospitalization for the diagnosis and subtyping of primary aldosteronism, patients were treated with drugs that have minimal influence on the renin–angiotensin–aldosterone system, such as non-dihydropyridine calcium-channel blockers, α-blockers, or direct vasodilators. At discharge, patients in the drug treatment group would be treated specifically with spironolactone and also with the guideline recommended antihypertensive drugs. The use of antihypertensive medications at discharge was significantly (*p* < 0.001) lower in the surgery than the drug treatment group, such as calcium channel blockers, angiotensin-converting enzyme inhibitors or angiotensin receptor blockers, and mineralocorticoid receptor antagonists. The mean dosage of the mineralocorticoid receptor antagonist spironolactone of 26 patients in the drug treatment group was 33.4 ± 12.9 mg/day during the whole follow-up period. The other two patients were with eplerenone with a dosage of 50 mg/day.

**Table 1:** Demographic and clinical characteristics at baseline.

Characteristic	Surgery (*n* = 39)	Drug treatment (*n* = 28)	*p*-value
Age (years)	49.4 ± 10.2	48.8 ± 11.5	0.830
Male sex, *n* (%)	26 (66.7)	22 (71.4)	0.286
Body mass index (kg/m^2^)	25.7 ± 3.3	26.8 ± 2.4	0.175
Systolic blood pressure (mmHg)	147.5 ± 13.2	144.1 ± 14.1	0.306
Diastolic blood pressure (mmHg)	85.7 ± 9.7	84.5 ± 7.8	0.576
Heart rate (beats/min)	68.0 ± 9.6	66.7 ± 8.8	0.597
Number of antihypertensive medications at admission	2.0 (2.0, 3.0)	3.0 (2.0, 3.0)	0.410
Serum creatinine concentration (μmol/L)	72.7 ± 13.3	80.9 ± 21.5	0.077
eGFR (ml/min*1.73 m^2^)	98.6 ± 14.3	94.2 ± 20.1	0.480
Serum potassium concentration (mmol/L)	3.3 ± 0.4	3.5 ± 0.4	0.036
24-h urinary potassium excretion (mmol)	74.1 ± 26.7	65.3 ± 25.6	0.178
Plasma aldosterone concentration (pg/ml)	368 (271, 494)	318 (252, 539)	0.469
Plasma renin activity (ng/ml/h)	0.27 (0.14, 0.47)	0.84 (0.31, 1.2)	<0.001
24-h urinary aldosterone excretion (μg)	26.7 (15.5, 35.0)	20.7 (15.5, 27.0)	0.021
Use of antihypertensive drugs at discharge, *n* (%)			
Calcium-channel blocker	13 (33.3)	24 (85.7)	<0.001
ACEI or ARB	11 (28.2)	25 (89.3)	<0.001
α-blocker	5 (12.8)	3 (10.7)	0.999
β-blocker	4 (10.3)	6 (21.4)	0.299
Mineralocorticoid receptor antagonist	0 (0)	28 (100.0)	<0.001

Values are mean ± SD, median (interquartile range), or percentage of patients (%). eGFR, estimated glomerular filtration rate, was calculated using the modified equation of the simplified Modification of Diet in Renal Disease (MDRD) formula (17).

Patients in the two groups had comparable echocardiographic measurements including global longitudinal strain and left ventricular pressure-strain loop parameters (**Table 2**).

**Table 2:** Echocardiographic measurements at baseline.

Variable	Surgery (*n* = 39)	Drug treatment (*n* = 28)	*p*-value
LAVI (ml/m^2^)	26.3 ± 6.3	26.7 ± 7.4	0.830
LVMI (g/m^2^)	108.3 ± 18.3	110.0 ± 20.5	0.725
Relative wall thickness	0.42 ± 0.06	0.42 ± 0.04	0.933
LVEDV (ml)	84.3 ± 23.4	80.3 ± 20.1	0.471
LVEF (%)	66.9 ± 4.5	66.0 ± 4.3	0.407
E (cm/s)	76.3 ± 16.1	76.3 ± 14.9	0.879
E/A	1.0 ± 0.3	1.0 ± 0.3	0.809
E/E’	9.1 ± 2.5	9.3 ± 2.3	0.572
Tei index	0.54 ± 0.11	0.58 ± 0.10	0.077
GLS (-%)	18.3 ± 2.7	18.4 ± 2.3	0.933
Left ventricular pressure-strain loop			
GWI (mmHg%)	2,372 ± 388	2,335 ± 341	0.681
GCW (mmHg%)	2,510 ± 360	2,437 ± 293	0.554
GWW (mmHg%)	201 ± 87	164 ± 56	0.097
GWE (%)	91.5 ± 3.1	92.5 ± 2.2	0.289

Values are mean ± SD. A, the peak atrial filling velocity of transmitral flow; E’, the average peak early filling velocity of septal and lateral mitral annulus; GCW, global constructive work; GLS, global longitudinal strain; GWE, global work efficiency; GWI, global myocardial work index; GWW, global wasted work; IVS, interventricular septum thickness; LAVI, left atrial volume index; LVEDD, left ventricular end-diastole diameter; LVEDS, left ventricular end-systole diameter; LVEF, left ventricular ejection fraction; LVMI, left ventricular mass index; LVPW, left ventricular posterior wall thickness.

### Biochemical Measurements and Blood Pressure During Follow-Up

In both surgery and drug treatment groups, PAC (83 [57, 119] and 231 [187, 432] pg/ml, *p* < 0.001 and *p* = 0.005, respectively) and 24-h urinary potassium excretion (52.0 ± 22.2 and 43.6 ± 8.3 mmol, *p* = 0.003 and 0.006, respectively) significantly decreased from baseline, and PRA (0.94 [0.52, 2.1] and 1.1 [0.49, 3.1] ng/ml/h, *p* < 0.001 and *p* = 0.001, respectively) and serum potassium concentration (4.4 ± 0.3 and 4.0 ± 0.3 mmol/L, *p* < 0.001 for both, respectively) significantly increased from baseline (**Table 3**). The 24-h urinary aldosterone excretion decreased significantly (*p* < 0.001) in the surgery group (2.7 [1.4, 5.0] μg), but not the drug group (12.8 [10.1, 16.8] μg, *p* = 0.057). The between-treatment differences reached statistical significance for the changes in PAC, serum potassium concentration, and 24-h urinary aldosterone excretion (*p* < 0.001). Nonetheless, the normalization of serum potassium concentration was achieved in almost all patients except one in the drug treatment group because of diarrhea the other day of the biochemical measurement (3.48 mmol/L).

**Table 3:** Changes in blood and urine biochemical measurements and in blood pressure and heart rate during follow-up.

Variable	Surgery	Drug treatment	Between-treatment difference (95% CI)	*p*-value
Blood measurements	*n* = 39	*n* = 25		
Plasma aldosterone concentration (pg/ml)	−347 ± 19*	−146 ± 24*	201 (140, 262)	<0.001
Plasma renin activity (ng/ml/h)	1.48 ± 0.34*	1.11 ± 0.41*	−0.37 (−1.50, 0.76)	0.513
Serum potassium concentration (mmol/L)	1.08 ± 0.07*	0.57 ± 0.08*	−0.51 (−0.71, 0.30)	<0.001
Urine measurements	*n* = 31	*n* = 20		
24-h urinary aldosterone excretion (μg)	−21.3 ± 0.93*	−9.7 ± 1.2	11.6 (8.5, 14.8)	<0.001
24-h urinary potassium excretion (mmol)	−16.1 ± 3.4*	−23.2 ± 4.0*	−8.1 (−18.8, 2.6)	0.134
Blood pressure and heart rate	*n* = 39	*n* = 28		
Systolic blood pressure (mmHg)	−9.1 ± 2.0*	−10.3 ± 2.4*	−1.2 (−7.5, 5.2)	0.710
Diastolic blood pressure (mmHg)	−1.7 ± 1.3	−3.4 ± 1.6	−1.8 (−5.9, 3.4)	0.390
Heart rate (beats/min)	5.3 ± 1.5*	3.7 ± 1.7	−1.5 (−6.0, 3.0)	0.501

Values per group are least square mean change from baseline ± SEM. Changes were computed by subtracting the values at baseline from the values at the last follow-up visit. Negative values indicate decrease from baseline. CI, confidence interval.

*p < 0.05 vs. baseline.

**Figure 2:**
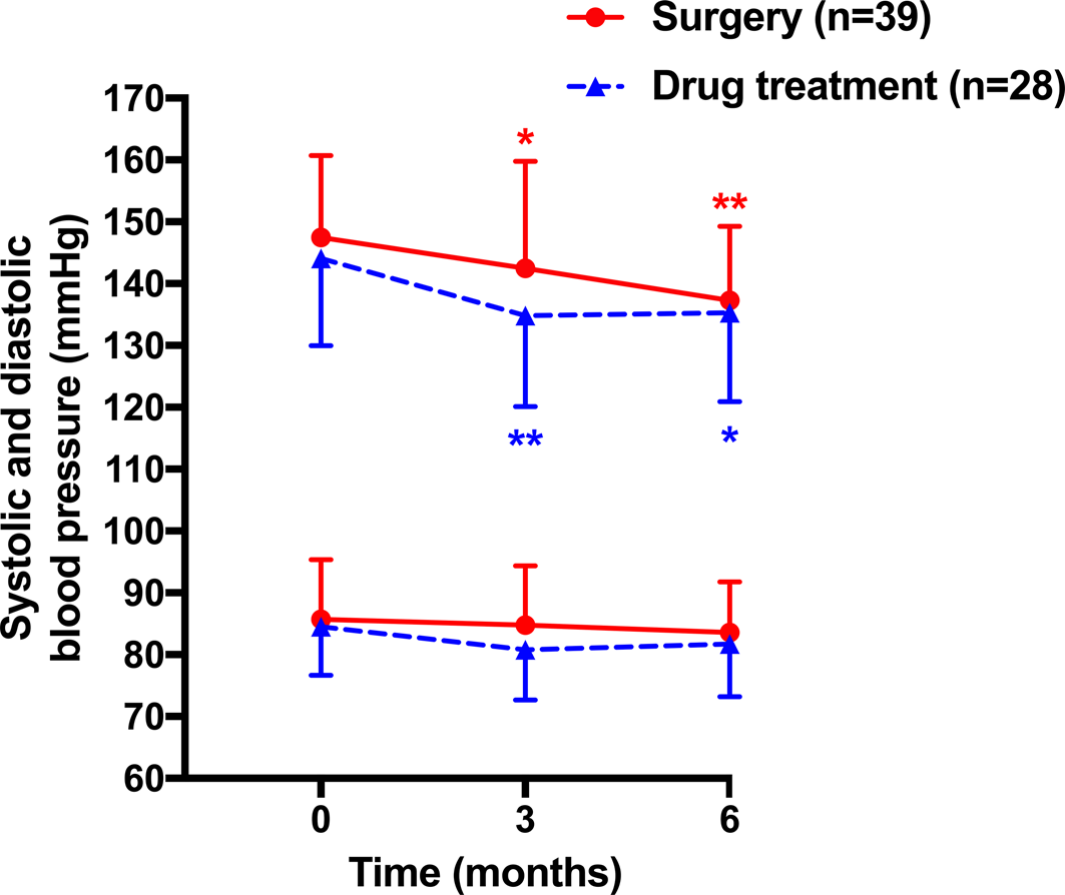
Mean (± SD) values of systolic (upper lines) and diastolic blood pressure (lower lines) at baseline and during follow-up in patients treated with surgery (circles with solid lines) and drugs (triangles with dashed lines). *p < 0.05, **p < 0.01 *vs*. baseline.

The changes in blood pressure reached statistical significance for systolic blood pressure at 3 (−4.4 ± 2.3 in surgery and −10.1 ± 2.7 mmHg in drug treatment group, *p* = 0.047 and 0.004, respectively) and 6 months (−9.1 ± 2.0 and −10.3 ± 2.4 mmHg, *p* < 0.001 and *p* = 0.017, respectively) of follow-up in the surgery (142.5 ± 17.3 at 3 months and 137.3 ± 12.0 mmHg at 6 months, respectively) and drug treatment groups (134.8 ± 14.7 and 135.3 ± 14.4 mmHg, **Figure 2**). Diastolic blood pressure remained similarly in both surgery and drug treatment group during follow-up. The between-treatment differences in the changes in systolic and diastolic blood pressures at 3 and 6 months of follow-up were −5.7 mmHg (95% confidence interval [CI], −12.9, 1.5 mmHg, *p* = 0.117)/−3.4 mmHg (95% CI, −7.3, 0.50 mmHg, *p* = 0.086) and −1.2 mmHg (95% CI, −7.5, 5.2 mmHg, *p* = 0.710)/−1.8 mmHg (95% CI, −5.9, 3.4 mmHg, *p* = 0.390) and did not reach statistical significance. Besides, 22 patients (56.4%) in the surgery group had achieved clinic blood pressure (≤140/90 mmHg) at 6 months of follow-up. The control rate was comparable to that in the drug treatment group (64.2%, *n* = 18, *p* = 0.517).

### Echocardiographic Measurements During Follow-Up

**Figure 3:**
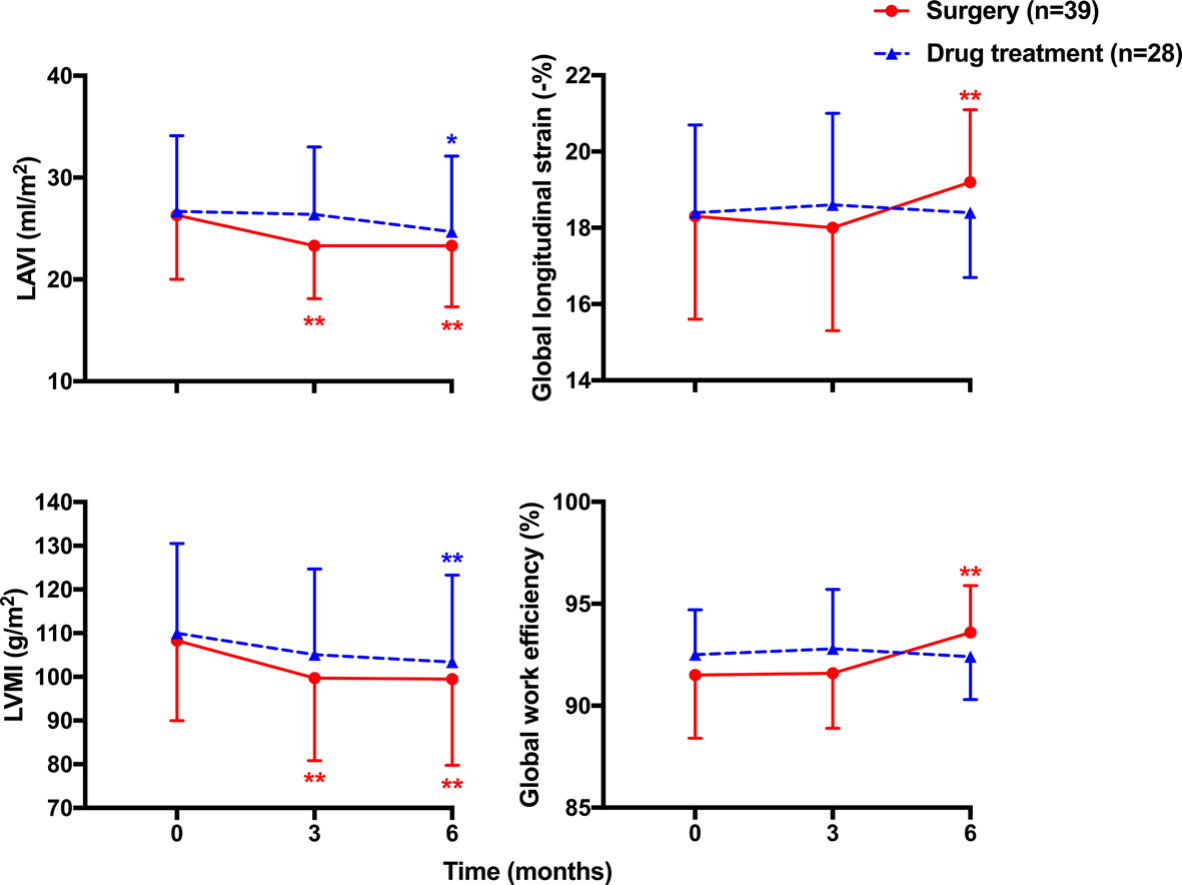
Mean (± SD) values of cardiac structure (left panels) including left atrial volume index (LAVI) and left ventricular mass index (LVMI), and function (right panels) including global longitudinal strain and global work efficiency of the left ventricle at baseline and during follow-up in patients treated with surgery (circles with solid line) and drugs (triangles with dashed line). *p < 0.05, **p < 0.01 *vs*. baseline.

Standard echocardiography showed significant changes in several structural and functional measurements at 6 months of follow-up, such as LAVI (*p* < 0.001 and *p* = 0.025, respectively), LVMI (*p* < 0.001 and *p* = 0.009, respectively), and E/E’ (*p* = 0.001 and 0.023, respectively) in both surgery and drug treatment groups, and LVEDV and peak E velocity in the surgery but not drug treatment group (**Figure 3** and **Table 4**). Statistical significance for the between-treatment differences was reached for the changes in LAVI (*p* = 0.001) and peak E velocity (*p* = 0.025) at 3 months and LVEDV at both 3 months (*p* = 0.004) and 6 months (*p* = 0.001) of follow-up (**Table 4**), all in favor of surgery.

**Table 4:** Changes in echocardiographic measurements at 3 and 6 months of follow-up.

	3 months				6 months			
Variable	Surgery (*n* = 39)	Drug treatment (*n* = 28)	Between-treatment difference (95% CI)	*p*-value	Surgery (*n* = 39)	Drug treatment (*n* = 28)	Between-treatment difference (95% CI)	*p*-value
Standard echocardiography								
LAVI (ml/m^2^)	−3.10 ± 0.54*	−0.18 ± 0.63	2.92 (1.27, 4.58)	0.001	−3.02 ± 0.67*	−1.97 ± 0.78*	1.06 (−1.00, 3.11)	0.307
LVMI (g/m^2^)	−8.7 ± 2.0*	−4.6 ± 2.4	4.1 (−2.0, 10.3)	0.184	−8.9 ± 1.9*	−6.4 ± 2.2*	2.5 (−3.3, 8.3)	0.390
Relative wall thickness	−0.001 ± 0.006	0.010 ± 0.008	−0.009 (−0.028, 0.011)	0.379	0.001 ± 0.007	−0.004 ± 0.009	−0.003 (−0.024, 0.017)	0.735
LVEDV (ml)	−13.3 ± 2.1*	−3.5 ± 2.5	9.8 (3.3, 16.4)	0.004	−15.2 ± 2.0*	−4.7 ± 2.4	10.5 (4.3, 16.7)	0.001
LVEF (%)	−1.07 ± 0.59	−0.02 ± 0.70	1.05 (−0.79, 2.89)	0.258	−0.31 ± 0.47	−0.39 ± 0.55	−0.08 (−1.52, 1.37)	0.917
E (cm/s)	−7.6 ± 2.1*	−0.27 ± 2.4	7.3 (0.94, 13.7)	0.025	−9.2 ± 1.8*	−5.3 ± 2.1	4.0 (−1.5, 9.4)	0.153
E/A	−0.019 ± 0.044	0.010 ± 0.052	0.009 (−0.127, 0.145)	0.896	−0.027 ± 0.037	−0.075 ± 0.044	−0.058 (−0.163, 0.067)	0.406
E/E’	−0.60 ± 0.26	−0.41 ± 0.31	0.18 (−0.63, 1.00)	0.654	−1.19 ± 0.23*	−0.72 ± 0.27*	0.48 (−0.24, 1.19)	0.190
Tei index	0.001 ± 0.017	−0.031 ± 0.020	−0.032 (−0.085, 0.020)	0.223	−0.025 ± 0.018	−0.017 ± 0.021	0.009 (−0.046, 0.064)	0.749
Strain echocardiography								
GLS (-%)	−0.33 ± 0.28	0.21 ± 0.33	0.54 (−0.34, 1.41)	0.227	0.89 ± 0.21*	0.04 ± 0.25	−0.85 (−1.51, −0.20)	0.011
Left ventricular pressure-strain loop								
GWI (mmHg%)	−186 ± 47*	−171 ± 55*	15 (−129, 159)	0.841	−83 ± 46	−140 ± 55*	−57 (−200, 87)	0.433
GCW (mmHg%)	−188 ± 48*	−167 ± 57*	21 (−127, 169)	0.778	−57 ± 47	−131 ± 55	−74 (−220, 72)	0.314
GWW (mmHg%)	−13.2 ± 8.7	−20.9 ± 10.3	−7.7 (−35.0, 19.5)	0.571	−50.4 ± 7.1*	−10.9 ± 8.5	39.5 (17.1, 62.0)	0.001
GWE (%)	−0.06 ± 0.36	0.54 ± 0.43	0.60 (−0.53, 1.73)	0.293	1.83 ± 0.30*	0.20 ± 0.35	−1.64 (−2.56, −0.71)	0.001

Values per group are least square mean change from baseline ± SEM. The between-treatment difference was calculated by subtracting the changes from baseline in the surgery group from those in the drug treatment group. Negative values of the between-group difference indicate greater reduction in the drug treatment group than surgery group. A, the peak atrial filling velocity of transmitral flow; CI, confidence interval; E’, the average peak early filling velocity of septal and lateral mitral annulus; GCW, global constructive work; GLS, global longitudinal strain; GWE, global work efficiency; GWI, global myocardial work index; GWW, global wasted work; LAVI, left atrial volume index; LVEDV, left ventricular end-diastole volume; LVEF, left ventricular ejection fraction; LVMI, left ventricular mass index.

*p < 0.05 vs. baseline.

Strain echocardiography showed similar decreases in GWI and GCW in both surgery and drug treatment groups at 3 months (*p* = 0.841 and 0.778, respectively) and 6 months of follow-up (*p* = 0.433 and 0.314, respectively). However, the changes in global longitudinal strain, GWW, and GWE tended to be greater in the surgery than the drug treatment group, especially at 6 months of follow-up. In line with the significant increases in global longitudinal strain in the surgery (*p* = 0.003) but not drug treatment group (*p* = 0.946), GWW significantly decreased and GWE significantly increased in the surgery (*p* < 0.001) but not the drug treatment group at 6 months of follow-up. The corresponding between-treatment differences were −0.85% (95% CI, −1.51, −0.20%, *p* = 0.011), 39.5 mmHg% (95% CI, 17.1, 62.0 mmHg%, *p* = 0.001), and −1.64% (95% CI, −2.56, −0.71%, *p* = 0.001), in favor of surgery (**Figure 3** and **Table 4**).

**Figure 4:**
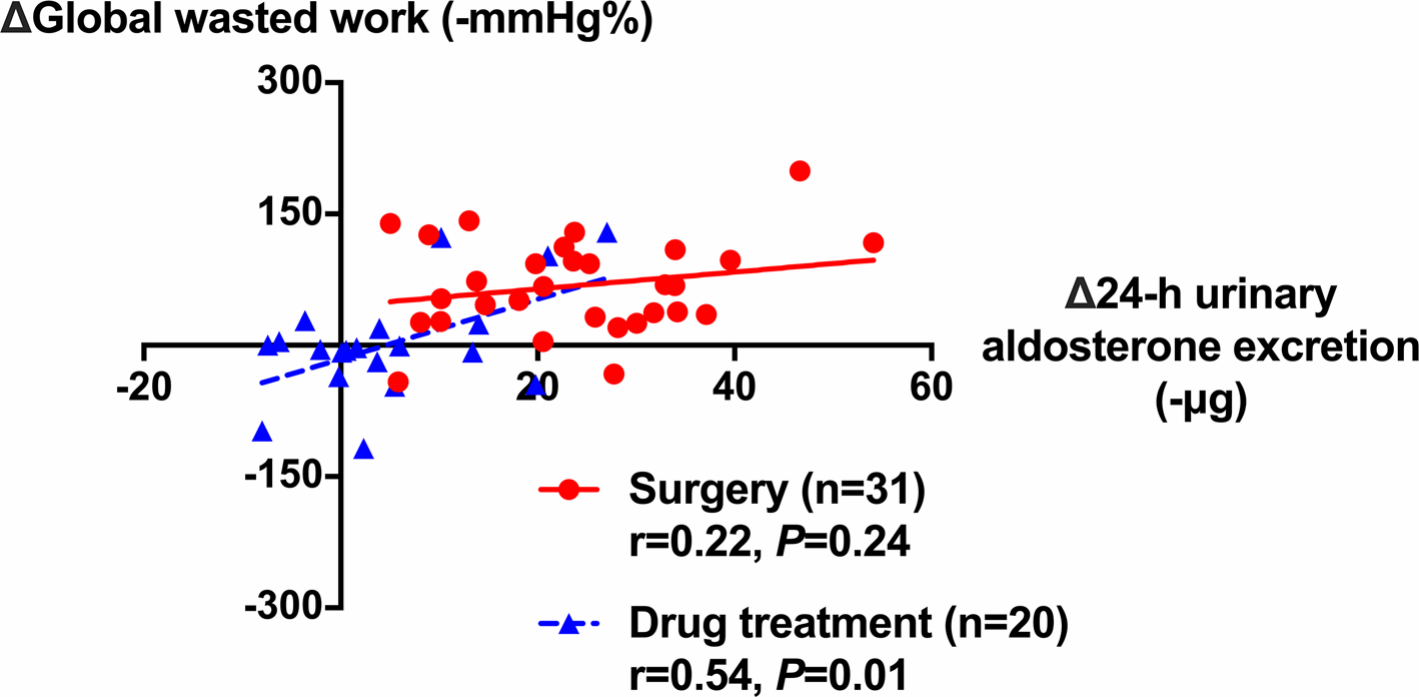
Correlation between the changes in global wasted work of the left ventricle and that in 24-h urinary aldosterone excretion during follow-up in patients treated with surgery (circles with solid line) and drugs (triangles with dashed line). The correlation coefficients and corresponding *p*-values are given for each group.

Further analyses showed significant correlation between the changes from baseline in GWW and that in 24-h urinary aldosterone excretion in the drug treatment group (*r* = 0.54, *p* = 0.014), and in the two groups combined (*r* = 0.55, *p* < 0.001), but not in the surgery group (*r* = 0.22, *p* = 0.242, **Figure 4**). In addition, the changes in global longitudinal strain were weakly correlated with age in the surgery group (*r* = 0.29) with a border significance (*p* = 0.075), but not in the drug treatment group (*p* = 0.983). There was no correlation between the changes in GWE or GWW and age.

### Intra- and Inter-Observer Variability of Myocardial Work Parameters

The analysis for the intra- and inter-observer variability for myocardial work parameters is presented in **Table 5**. The intra-class correlation coefficients ranged from 0.93 to 0.97 with the lower boundary of all 95% CI over 0.70.

**Table 5:** Intra- and inter-observer variability (*n* = 10).

Variable	ICC	95% CI	*p*-value
Intra-observer			
GLS (-%)	0.97	0.89–0.99	<0.001
GWI (mmHg%)	0.93	0.72–0.98	<0.001
GCW (mmHg%)	0.95	0.81–0.99	<0.001
GWW (mmHg%)	0.95	0.80–0.99	<0.001
GWE (%)	0.95	0.78–0.99	<0.001
Inter-observer			
GLS (-%)	0.97	0.88–0.99	<0.001
GWI (mmHg%)	0.95	0.81–0.99	<0.001
GCW (mmHg%)	0.97	0.89–0.99	<0.001
GWW (mmHg%)	0.93	0.70–0.98	<0.001
GWE (%)	0.96	0.85–0.99	<0.001

Values are intra-class correlation coefficient (ICC) with the 95% confidence interval (CI) and p-value.

GCW, global constructive work; GLS, global longitudinal strain; GWE, global work efficiency; GWI, global myocardial work index; GWW, global wasted work.

## Discussion

Our study demonstrated that the surgical treatment with adrenalectomy had an effect of early regression of cardiac structure and definite improvement of cardiac function, although both surgery and drug treatment significantly reduced blood pressure and normalized serum potassium concentration. The significant correlation between the changes in cardiac function and aldosterone level in the drug treatment but not surgery group suggested that adrenalectomy might have treatment effects over and beyond reductions in aldosterone level and mineralocorticoid receptor antagonism.

To the best of our knowledge, this is the first study that has applied the non-invasive left ventricular pressure-strain loop technique to compare cardiac structural and functional alterations in primary aldosteronism after various treatments. A unique feature of this novel technique is the incorporation of left ventricular pressure with the measurements of cardiac deformation, allowing detection of cardiac physiological and functional changes under various afterloads in patients with cardiovascular diseases (18, 19). The wasted work might act as an index of energy loss to indicate dyssynchrony and remodeling (20), while the constructive work and myocardial work index are more related to the total energy and oxygen consumption at various afterloads (21). Indeed, we observed significant decreases in GWI and GCW during follow-up probably due to the lowering of blood pressure, and a significant decrease in GWW and increase in GWE in the surgery group, suggesting an improvement in left ventricular remodeling and resynchronization. Previous studies showed cardiac structural alterations after both surgery and drug treatment. In a prospective study, Lin et al. found significant regression of LVMI (163 ± 49 at baseline *vs*. 128 ± 38 g/m^2^ during follow-up, *n* = 20, *p* = 0.01) in patients with adrenalectomy at 1 year of follow-up (7). In a retrospective study of drug treatment with a mean dosage of spironolactone of 33.3 ± 13.7 mg/day, Ori et al. found that LVMI significantly decreased at 1 year of follow-up (142.9 ± 25.4 *vs*. 117.7 ± 20.4 g/m^2^, *n* = 48, *p* < 0.001) and normalized at 3 years (22). Few studies investigated cardiac systolic function because of difficulties in the detection of early cardiac changes by standard echocardiography. In our study, the similar and significant cardiac structural alterations in LVMI and LAVI in both groups might be related to the similar decreased afterload in blood pressure, while the significant decrease in preload assessed as LVEDD only in the surgery group could additionally contribute to the improvement of cardiac function evaluated by the novel non-invasive echocardiographic method. Besides, the possibility of regression in cardiac fibrosis in the surgery group might also play a part as it was confirmed in a previous study in post-adrenalectomized patients by myocardial biopsy (23).

The effectiveness of blood pressure lowering and potassium normalization after both treatments corroborated the findings of previous studies on post-treatment clinical and biochemical changes. The only hypokalemia because of diarrhea was normalized to 4.34 mmol/L at the regular follow-up visit after the end of our study without medication changes. Rossi et al. found in a prospective study that both systolic and diastolic blood pressure decreased significantly (*p* < 0.01) after surgery (164 ± 20 at baseline *vs*. 137 ± 15 mmHg during follow-up, 100 ± 12 *vs*. 83 ± 9 mmHg, *n* = 110, respectively) and drug treatment (165 ± 21 *vs*. 133 ± 11 mmHg, 101 ± 12 *vs*. 83 ± 7 mmHg, *n* = 70, respectively) during a median follow-up of 36 months along with a significant increase in serum potassium concentration in both groups (3.19 ± 0.56 *vs*. 4.12 ± 0.44 mmol/L in the surgery group, 3.33 ± 0.51 *vs*. 4.00 ± 0.44 mmol/L in the drug treatment group, *p* < 0.01, respectively) (24). In a short-term retrospective study with 6 months of follow-up, Katabami et al. found similar decreases in blood pressure and serum potassium normalization rate in age- and gender-matched patients with surgical and medical treatments (25).

Several potential mechanisms might explain why both surgery and drug treatment effectively improved clinical and biochemical changes, but only surgery, but not drug treatment, improved cardiac function beyond the changes in aldosterone level. First, sufficient inhibition of aldosterone in short-term could only be achieved in those surgically treated patients, because unilateral adrenalectomy could fully remove the cause of hyperaldosteronism while mineralocorticoid receptor antagonists might take a much longer time to show any effect of inhibition (26). Indeed, in a prospective long-term study with an average follow-up of 6.4 years, Catena et al. found that the response of LV mass occurred earlier in adrenalectomized patients than in those treated with spironolactone but later became comparable in the two groups of patients (27). Second, adrenal hormones other than mineralocorticoid might have decreased in patients with adrenalectomy, such as cortisol. Autonomous secretion of cortisol was found in patients with primary aldosteronism in previous studies and might have an impact on cardiac structure and function (28, 29), and possibly further increase the risk of cardiovascular events (30). Other mechanisms might also play a part, such as the novel mineralocorticoid receptor-independent pathway mediated by G protein-coupled estrogen receptors (31). Caroccia et al. found that aldosterone stimulated its biosynthesis to exert rapid nongenomic effects through this novel mechanism other than classic activation of mineralocorticoid receptor, which could not be blocked by spironolactone (32). Besides, a weak positive correlation between the changes in global longitudinal strain and age was found in the surgery group. There is currently very limited published data in this particular regard. Nonetheless, the results seem to indicate greater cardiac benefit of adrenalectomy with age advancing, probably because older patients, compared with younger ones, had longer duration of hypertension and hyperaldosteronism and more severe cardiac damage and were more likely to have treatment-induced changes. Interestingly, PAC decreased significantly in both surgery and drug treatment group though usually the level might increase in response to mineralocorticoid receptor antagonism. Similar results were found in a study of Rossi et al. (24). Their explanation was that the decrease was probably induced by the treatment with renin–angiotensin system antagonists. Indeed, most of study participants were treated with these antihypertensive agents.

Our study has several limitations. First, our study was prospective but had an observational, instead of randomized controlled trial, design, and did not include a group of healthy control subjects. The sample size was quite small, especially for the biochemical measurements. Second, though matched for several important characteristics, it should be noted that plasma renin activity was significantly lower and 24-h urinary aldosterone excretion was significantly higher in the surgery than the drug treatment group. This might suggest more severe hyperaldosteronism in the surgery than the drug treatment group and could possibly impact the final results of our study, as cardiac improvement might be more prominent in patients with more severe hyperaldosteronism. Third, although blood pressure decreased significantly and serum potassium concentration normalized in the drug treatment group, the low dose of spironolactone at 33.4 mg/day might be insufficient to induce significant cardiac alterations. Routinely, we titrate the dose of spironolactone primarily based on the serum potassium level. This raises a question on the dose-titration approach of mineralocorticoid receptor antagonism in primary aldosteronism. Fourth, arterial systolic pressure measured with a cuff in the brachial artery can be higher than the left ventricular systolic pressure and hence less accurate than the invasive left ventricular pressure measurements. We excluded patients with aortic stenosis, left ventricular outflow tract obstruction, and any other cardiac pathologies that may induce a pressure gradient between left ventricle and aorta. Fifth, this is a short-term prospective study. Long-term alterations on the cardiac structural and functional measurements with the new echocardiographic technique remain under investigation.

## Conclusion

In conclusion, only surgical removal of the adrenal gland showed significant improvement in cardiac structure and function, though both surgery and drug treatment effectively reduced blood pressure and normalized serum potassium, probably because of sufficient inhibition of aldosterone and other therapeutic effects of adrenalectomy.

## Data Availability Statement

The datasets presented in this article are not readily available because our dataset is only available to our research team. Requests to access the datasets should be directed to T-YX, xtyswallow@sina.com.

## Ethics Statement

The studies involving human participants were reviewed and approved by the Ethics Committee of Ruijin Hospital, Shanghai Jiao Tong University School of Medicine, Shanghai, China. The patients/participants provided their written informed consent to participate in this study.

## Author Contributions

Formal analysis: Y-LC and T-YX. Clinical data collection: J-ZX and L-MZ. Project administration: YL. Supervision: J-GW. Writing—original draft: Y-LC. Writing—review and editing: T-YX and J-GW. All authors contributed to the article and approved the submitted version.

## Funding

The present study was financially supported by the Shanghai Municipal Commission of Health (grant 201840064). Drs. Yan Li and Ji-Guang Wang were also financially supported by grants from the National Natural Science Foundation of China (grants 91639203, 81770455, 82070432, and 82070435), Ministry of Science and Technology (grants 2015AA020105-06 and 2018YFC1704902), Ministry of Health (grant 2016YFC0900902), Beijing, China, and the Shanghai Commissions of Science and Technology (grant 19DZ2340200), Education (Gaofeng Clinical Medicine Grant Support 20152503), and Health (grants 15GWZK0802 and 2017BR025 and a special grant for “leading academics”).

## Conflict of Interest

The authors declare that the research was conducted in the absence of any commercial or financial relationships that could be construed as a potential conflict of interest.

## Publisher’s Note

All claims expressed in this article are solely those of the authors and do not necessarily represent those of their affiliated organizations, or those of the publisher, the editors and the reviewers. Any product that may be evaluated in this article, or claim that may be made by its manufacturer, is not guaranteed or endorsed by the publisher.

## Abbreviations

ARR, aldosterone-to-renin ratio; GCW, global constructive work; GWE, global work efficiency; GWI, global myocardial work index; GWW, global wasted work; LAVI, left atrial volume index; LVEDV, left ventricular end-diastole volume; LVEF, left ventricular ejection fraction; LVMI, left ventricular mass index; PAC, plasma aldosterone concentration; PRA, plasma renin activity.
